# Health Promotion Behaviors and Chronic Diseases of Aging in the Elderly People of Iranshahr*- IR Iran

**DOI:** 10.5539/gjhs.v8n3p139

**Published:** 2015-07-13

**Authors:** Zahra Pishkar Mofrad, Mozhgan Jahantigh, Azizollah Arbabisarjou

**Affiliations:** 1 Pregnancy Health Research Center, Zahedan University of Medical Sciences, Zahedan, IR Iran

**Keywords:** health promotion behaviors, chronic diseases, aging

## Abstract

**Introduction and Aim:**

Aging is considered as the phenomenon of the day in the health arena of the world and Iran. It is anticipated that there will be an explosion of aging population in Iran in about 2031 and 20-25% of the population will be aged over 60 years. With aging, chronic diseases also increase and diminish the functional ability of older people. On the other hand, increased healthcare costs should be also added to this issue. Health promotion is a concept of process that continues throughout life. As much as health promotion is important in children and adults, it is equally important in older people. In fact, the elderlies, as a group, also acquire many benefits from health promotion behaviors. Due to the increasing elderly population, geriatric health promotion and enhancing the health level of older people is proposed as a health priority that should be properly planned. Hence, the present study has been conducted in this regard and aims to identify behaviors of health promotion and chronic diseases of aging in the elderly people of Iranshahr-Iran.

**Materials and Methods:**

The present research is a cross-sectional descriptive study whose population consists of 425 elderly people aged 60 years and over, who lived in the city of Iranshahr*, IR Iran. The random cluster sampling method has been used to select the research samples. The required information was collected using a questionnaire which was distributed among the older people through visiting their homes; then, the collected data was statistically analyzed using the statistical software of SPSS version 13.

**Findings:**

The research findings show that the mean age of older people is 66.33 ± 7.7 and the highest frequency belongs to the age group of 60 years and the maximum age is 92 years. 69.5% of the older people were in the age group of the young elderly (60-69 years) and 44% of them lived with their married children; also 55.8%, 81.9%, 70.5%, and 74.4% of them were respectively female, illiterate, married, and unemployed. The mean score of geriatric health promotion behaviors was 6.1 ± 1.87 in the range of 0-11 and 54.9% of them got the score of the inappropriate health promotion behavior. The most frequent chronic diseases of older people were respectively joint problems (78.9%), sensory problems (64.1%), and hypertension (56.6%). No significant relationship was observed between the score of health promotion behaviors and “the gender, lifestyle and marital status of the older people”, but there was statistically a significant relationship between the score of health promotion behaviors and “the age, education, and job of the older people”.

**Conclusion:**

Providing training programs for health promotion behaviors in elderly people can improve these behaviors and enhance their health and quality of life and reduce the incidence of chronic diseases in them. The goal of health promotion behaviors is to maintain performance, independence and quality of life; and many studies have demonstrated that the elderly people who aged 60 years and over benefit from health promotion behaviors more than middle-aged people.

## 1. Introduction

Aging is a critical period of human life and attention to the problems and needs of this stage is a social necessity. Aging is a natural, physiological, and time-dependent process, which begins at birth and continues until the end of life. In fact, aging is the last stage of the successive stages of life (Memarian, 1999; Timby & Smith, 2013). Terms such as elderly, aging, old, aged, and so on are usually used for those who have passed the age of 60 years or more (Tajvar, 2003). Life expectancy is the average number of years a person is expected to live, and has significantly increased in the last hundred years (Brunner et al., 2010). The fact is that life expectancy has increased globally and this issue has been followed by the elderly phenomena in communities (Franceschi & La Vecchia, 2001). The number of elderly people is rapidly increasing worldwide. The world population is currently 6.3 billion people and will reach almost 8.7 billion people by 2050, from which 2 billion people will be aged 60 years and older Population (Salar, Alireza, & Fazlollah, 2004).

In Iran, the Geriatric Health Department of the Ministry of Health and Medical Education has announced that the acceleration of aging has been significant in the country. Currently, Iran is in the transition from young population to an aging one and soon it will be also among the countries with aging populations. At present, elderly people constitute 6.8% of the Iranian population and this rate will increase to 9 to10 million of the total population of the country (namely 10% of it) by 2030 (Teymoori et al., 2006). Iran will be place at superior ageing phase in thirty years in future. The highest pick of ageing is allocated to Iran in the world. Due to decreasing the children in the Iran, Iran has entered in to ageing phase with % 2.8 of geriatric population. Now, Iran has entered into the firat of ageing phase means %7-14 of geriatric population. It is predicted that Iran will entered into superior-ageing phase in next thirty years (more than 21% aged population) (Iran-newspaper, 2012). According to the censuses, the population of Sistan and Baluchestan Province has been 1,722,579 and 2,405,742 people in October 1996 and October 2006, respectively; from which 84,467 (in 1996) and 105,106 (in 2006) people were the adult population aged 60 years and older. Iranshahr is the oldest and largest city of Sistan and Baluchestan Province and its inhabitants are mostly indigenous and Balouch. According to the census taken in 2006, the population of the county and city of Iranshahr has been respectively 268,400 and 117,476 people, from which 11,473 and 3037 people were respectively the adult population aged 60 years and older in the county and city of Iranshahr. As the statistics of the country show, the city of Iranshahr has the youngest population among the cities of Iran (Housing, 1996).

According to the Pender's theory, health promotion behaviors include any action that is performed to increase or maintain the individual or group health and self-actualization (Richter et al., 1987). Health promotion behaviors are considered as one of the main criteria for determining health and preventing from being affected by many known diseases. In fact, health promotion and the prevention of disease are directly related to these behaviors (Loeb, 2003). Health promotion is a concept of process that continues throughout life. According to studies, a child's health can be positively or negatively affected by the mother's health habits during prenatal period; therefore, health promotion begins before birth and continues throughout childhood, youth, middle age, and old age (Brunner et al., 2010). Among the behaviors that must be encouraged and confirmed to reduce the loss of life due to premature mortality and ensure more and better quality of the remaining years of life, it can be pointed to regular exercise, adequate sleep, avoiding alcohol and tobacco use, proper nutrition, avoidance of obesity, age-appropriate vaccinations, medical care, and avoidance of stresses. As life expectancy increases, the importance of health promotion behaviors, with regard to maintaining functionality and independence of individuals and improving their quality of life, increasingly becomes apparent. Health promotion behaviors in elderly people have a potential impact on promoting their health and quality of life and equally reduce costs related to health care (Rocha et al., 2002).

One reason for the upward trend of chronic diseases in the world is the increase of life expectancy (Park, 2000). Aging is associated with a significant and higher prevalence of acute and chronic diseases as well as an increase in practical dependence on others. The results of the study conducted by Barry (2009) showed that 80% of elderly people have at least one chronic disease such as arthritis, blood pressure, respiratory diseases, cardio-vascular and sensory disorders (Barry, 2000). The majority of deaths (75%) in the United States occur in people aged 65 years and older, and more than half of the deaths are due to chronic diseases such as heart diseases and cancers (Brunner et al., 2010).

The increasing number of elderly people in the society increases the need for disease prevention and health promotion services, because the increase in the elderly population rises the risk of chronic diseases such as diabetes, cardiovascular, joint and bone diseases. These services help to maintain the maximum ability of an elderly person to be independence and get involved in the society as an active member and have the best quality of life (Salar et al., 2004). Also, elderly people are at the highest risk for lifestyle-related diseases such as heart diseases; hence, they will benefit more from health promotion behaviors (Levy et al., 2006). Due to the increasing elderly population, geriatric health promotion and enhancing the health level of older people is proposed as a health priority that should be properly planned, because many problems of old age are caused by the unhealthy lifestyle in this period (Saffari & Fariba, 2006).

Since the population structure of Iran is still young, the aging problem has not become severe yet; however, it is expected that in the coming years, the country is heavily involved with the problem; therefore, from now on, it is necessary to take a comprehensive plan for teaching the healthy lifestyle in old age. To achieve this goal, it is necessary to know the exact situation and needs of this population group. Now considering the fact that in the next few years, elderly adults will constitute a great proportion of Iran, if these people are healthy, they will not be only a consumer. In fact, the lower their physical problems and chronic diseases are, the lower their costs will be. In this way, the two goals including the reduction of treatment costs and improving the quality of life of elderly people will be achieved. Accordingly, the present study attempts to investigate the health promotion behaviors of the elderly people of Iranshahr-Iran. Hence, the findings of this research can provide useful guidance for health policy makers of the region to develop appropriate plans for the population group of this Province. Thus, the present study is aimed at identifying health promotion behaviors and common chronic diseases of the elderly people of Iranshahr-Iran.

## 2. Materials and Methods

The present research is a cross-sectional descriptive study aimed at investigating behaviors of health promotion and chronic diseases of aging in the elderly people of Iranshahr-Iran. The research population consisted of all elderly people aged 60 years and over, who were mentally healthy and lived in the city of Iranshahr at the time of data collection. After a pilot study on a limited number of research units and using statistical formulas based on the calculated standard deviation, they were 425 elderly aged people, from which the samples were selected using the random cluster sampling method; in this way that two clusters were selected from each health center in the city of Iranshahr. Since there are seven health centers in this city, 14 clusters were totally selected. From each cluster, thirty families with elderly members were chosen. All elderly members of these families were enrolled and it made no difference how many of them lived in each selected family. Numbers of clusters were selected randomly and far apart. The required information was collected using a questionnaire which was distributed among the elderly people through visiting their homes. In addition of the questionnaire, a researcher-made checklist was also used as data collection instrument. The tools were developed using similar articles and authoritative sources and included three parts: 1- demographic data (age, gender, education, lifestyle, marital status, employment, etc.), 2- a list of chronic diseases, and 3- health promotion behaviors.

The items of the questionnaire were scored in this way that the elderly person received a score for performing each health promotion behavior. The maximum score that a person can obtain eleven. In the case of receiving more than half of the score, health promotion behaviors are considered for the elderly person. After random selection of clusters and visiting the subjects, all females and males whose birth date, according to their national ID card, was before September 1947, namely, they were 60 years and over, entered the research population. Then, the reason of visiting them was briefly explained to them; and after getting their consent and ensuring the confidentiality of information, the questionnaire was delivered to the person for being completed. In the case of illiteracy or visual problems of subjects, the items of the questionnaire were read by the researcher and their answers were inserted in the questionnaire. All females and males who were 60 years old and over and enjoyed alertness and mental health and were able to hear and talk, were included in the statistical population, but the people with psychological disorders, deafness or speech disabilities were excluded from the population.

The statistical software of SPSS (version 13) along with the statistical tests including correlation test, Tukey test, Chi-square, t-test, analysis of variance at the confidence interval of 95% were used to analyze the research data.

Ethics Committee of Zahedan University of Medical Sciences has approved this research.

## 3. Findings

According to findings, the mean age of elderly people was 66.33 ± 7.7 and the highest frequency belonged to the age group of 60 years and the maximum age is 92 years. Majority of the elderly people (69.5%) included in the age group of the young elderly (60-69 years) and 44% of them lived with their married children. Also 55.8%, 81.9%, 70.5%, and 74.4% of them were respectively female, illiterate, married, and unemployed. In this study, the frequency of health promotion behaviors of elderly people for behaviors of non-smoking, suitable exercise, low-salt diet, low-fat diet, eating at least three meals of fish per week, eating up to 5 meals of red meat per week, blood pressure control, control of the health status, and the flu vaccine injection in the past year are respectively 70.4%, 69.7%, 48.5%, 46.7%, 40.6%, 59.1%, 60.9%, 47.5%, and 8.6%. The mean score of geriatric health promotion behaviors was 6.1 ± 1.87 in the range of 0-11 and 54.9% of them got the score of the inappropriate health promotion behavior. The most frequent chronic diseases of elderly people were respectively joint problems (78.9%), sensory disabilities (64.1%), and hypertension (56.6%). No significant relationship was observed between the score of health promotion behaviors and “the gender, lifestyle and marital status of the elderly people”, While there was statistically a significant relationship between the score of health promotion behaviors and “the age, education, and job of the elderly people”.

The analysis of variance was used to compare the scores of health promotion behavior of elderly age groups. The findings showed that there was statistically a significant difference (P=0.003) between elderly age groups in terms of the mean of health promotion behaviors (Table 1). The results of Tukey's post hoc test showed that the mean score of the young elderly group was higher than the elderly and older elderly groups, but there was no significant difference between the elderly and older elderly groups in this regard.

**Table 1:** The comparison of the mean score of health promotion behaviors in elderly age groups of Iranshahr

Variable		Health Promotion Behavior Score			

		Number	Mean	Standard Deviation	Statistical Analysis
Age Groups	Young Elderly	294	6.30	1.87	F= 5.912
	Elderly	91	5.75	1.81	Df=420
	Older Elderly	38	5.42	1.74	P=0.003 ANOVA

Using the independent t-test and comparing the scores of health promotion behaviors based on the history of chronic diseases showed statically a significant difference (P<0.0001) (Table 2). In other words, the elderly people with chronic diseases had higher scores of health promotion behaviors.

**Table 2:** The comparison of the mean score of health promotion behaviors in elderly people with chronic diseases

Variable		Health Promotion Behavior Score			

		Number	Mean	Standard Deviation	Statistical Analysis
Hypertension	Disease History	212	6.58	1.83	F= 5.516
	Not Affected	206	5.6	1.79	Df=416
					P<0.0001
Cardiovascular Disease	Disease History	132	6.37	1.91	T= 3.963
	Not affected	291	5.94	1.84	Df=412
					P=0.031
Hyperlipidemia	Disease History	130	6.94	1.66	T= 6.642
	Not affected	293	5.69	1.82	Df=421
					P<0.0001
Diabetes	Disease History	57	7	1.76	T= 2.158
	Not affected	357	5.95	1.85	Df=421
					P<0.0001

## 4. Discussion

According to the results, 55.8% of elderly people were female and 69.5% of them were in the age group of young elderly (60-69). In a study conducted on Korean elderlies, researchers have reported the frequency of female elderlies 70.2%. Also, according to this report 56.7% of elderlies were in the age group of 65-74 (Lee et al., 2006). Orfila et al., (2006) found similar results: a frequency of 65.4% for female elderlies. In developed countries, on average, women live six to eight years longer than men. This issue can lead to an increase in the gap between the gender and age. It should be noted that according to the censuses taken in 2006, in Sistan and Baluchestan province and most of its counties, especially in Iranshahr, the number of male elderlies are higher than females. The reasons may be related to the high number of pregnancies and high mortality rate of women due to the complications of pregnancy. Also, the results showed that 29.5% of elderly people have lost their spouse. In a research, 28.8% of elderly people in villages of Ardebil-Iran have lost their spouse (Satari, 2007). In this regard, Lee et al. (2006) have reported the frequency of such elderlies to be 47.8%. The difference between results of the studies conducted by Sattari and Lee et al. is resulted from the difference between Korean and Iranian societies in terms of culture and religious beliefs about the second marriage and life expectancy in the elderly population. It should be noted that the second marriage and multiplicity of wives may be one of the main reasons for such differences between Iranian and foreign studies in this regard.

In this study, 82% of elderly people were illiterate. Ahmadi et al. (2004) in a study conducted on the elderly people of Zahedan-Iran have reported the percentage of illiteracy to be 80.5%. The reason for the similarity of results is that the both regions under study, namely Iranshahr and Zahedan are two cities of Sistan and Baluchestan province which is a poor province and the majority of its elderly population has been illiterate or poorly educated in the past decades. 7.3% of elderly people live alone or with their relatives and the rest live with their spouse or married or single children; in other words, 92.7% of elderly people somehow live with their families. This can have origin in cultural, traditional context, their commitment to the values and norms of society, honoring and supporting the elderlies. In the study conducted by Saffari et al., 2006) the percentage of elder people living alone has been reported to be 8.6% while Lee et al.(2006) have reported the percentage of such elderlies to be 32.8% (Lee et al., 2006; Satari, 2007; Saffari et al., 2006). Again, the reason for the difference between the results of these two studies is the difference between the culture and religious beliefs of the two societies.

In the study of Lee et al. (2006) the mean score of health promotion behaviors has been reported equal to 3.97 ± 1.34 (ranging from 0 to 7). The findings show that the frequency of health promotion behaviors of non-smoking, low-salt diet, low-fat diet, eating up to 5 meals of red meat per week, and control of blood pressure, sugar and lipids was higher in female elderlies. Orfila et al. (2006) showed in their study that 23.47% of men and 93.8% of women had never been smoked. The reason for the difference between the frequency of men and women in terms of the behavior of smoking is that the women of this region mostly smoke hookah.

It has reported the frequency of chronic diseases in the elderly population studied by them as 62.3%, 33.8%, 20.2%, 15.6%, 37.7%, and 41.7% for arthritis, blood pressure, heart diseases, diabetes, cataracts, and hearing loss, respectively (Orfila et al., 2006). Smeltzer has reported the frequency of most common chronic diseases in the population studied by her as 58.5%, 52.6%, and 42.7% for high blood pressure, arthritis, and high blood lipids and cholesterol, respectively (Brunner et al., 2010).

1.4% of the elderly people under study have reported no history of chronic disease. According to Barry, 80% of elderly people have at least one sensory chronic disease such as arthritis, high blood pressure, cardiovascular diseases, or sensory disorders (Barry, 2000). In this study, 78.4% of elderlies have reported four or more chronic diseases. At least 75% of elderlies are aged 65 years and older had a chronic disease. Approximately 50% of them were suffering from at least two chronic diseases (Kass-Bartelmes, 2002).

The incidence of diseases such as hypertension, cardiovascular diseases, hyperlipidemia, and diabetes are closely related to the lifestyle of people. The results of studies show that the majority of elderly patients with chronic diseases perform most health promoting behaviors such as non-smoking, physical activity, low salt and low fat diet, and annual check of blood pressure, blood glucose and lipids; or in other words, the elderly patients with these diseases perform more health promotion behaviors in comparison with other elderly people. Since this study only investigates health promotion behaviors of elderly people at the present time and does not deal with the behaviors throughout their life, the high frequency of these behaviors in this study can be due to incidence of the diseases and doctors’ recommended treatment that have made the elderlies to perform health behaviors. The study conducted by McPhee et al. show that elderly adults who had reported higher health problems follow higher healthy lifestyle habits compared to adults without problems and diseases. Usually, people start a larger number of health habits when they notice the presence of specific and serious health problems (Brunner et al., 2010).

The results show that the mean score of health promotion behaviors in elderly females is higher than males; however, the difference between the two genders was not statistically significant. Lee et al. (2006) have reported a significant difference between the score of health promotion behaviors and gender.

Also, the results show that the highest frequency of performing health promotion behaviors is related to the age group of young elderlies. In the study conducted by Lee et al., 56.7% of subjects were in the age group of 65-74 and reported the highest frequency of health promotion behaviors. In addition, the mean score of health promotion behaviors was higher in young elderlies and the difference between young elderlies and older ones was significant in this regard. In other words, there is statistically a significant difference between the mean score of health promotion behaviors and the age group of elderlies (Lee et al., 2006). The reason for higher score of health promotion behaviors in the younger age group is that the younger people generally have greater life expectancy, higher functional ability, and lower chronic diseases.

Regarding the relationship between health promotion behaviors and literacy, the results showed that the frequency of these behaviors is higher in literate elderlies and there is statistically a significant difference between the education and the score of health promotion behaviors, so that the mean score of health promotion behaviors in educated elderlies is higher than the illiterate ones. These results are consistent with results of the study conducted by Lee et al. (2006).

## 5. The Conclusion

Providing training programs to promote health behaviors in elderly people can improve these behaviors and increase their health and quality of life and reduce the incidence of chronic diseases in them. The goal of health promotion behaviors is to maintain performance, independence and quality of life; and many studies have demonstrated that the elderly people who aged 60 years and over benefit from health promotion behaviors more than middle-aged people.
